# Carbon dot-based nanomaterials: a promising future nano-platform for targeting tumor-associated macrophages

**DOI:** 10.3389/fimmu.2023.1133238

**Published:** 2023-05-02

**Authors:** Yingying Miao, Shuang Wang, Butian Zhang, Lin Liu

**Affiliations:** Department of Radiology, China-Japan Union Hospital, Jilin University, Changchun, Jilin, China

**Keywords:** TAMs, carbon dots (CDs), bioimaging, theranostics, macrophage modulation

## Abstract

The tumor microenvironment (TME) is the internal environment that tumors depend on for survival and development. Tumor-associated macrophages (TAMs), as an important part of the tumor microenvironment, which plays a crucial role in the occurrence, development, invasion and metastasis of various malignant tumors and has immunosuppressant ability. With the development of immunotherapy, eradicating cancer cells by activating the innate immune system has yielded encouraging results, however only a minority of patients show a lasting response. Therefore, *in vivo* imaging of dynamic TAMs is crucial in patient-tailored immunotherapy to identify patients who will benefit from immunotherapy, monitor efficacy after treatment, and identify alternative strategies for non-responders. Meanwhile, developing nanomedicines based on TAMs-related antitumor mechanisms to effectively inhibit tumor growth is expected to become a promising research field. Carbon dots (CDs), as an emerging member of the carbon material family, exhibit unexpected superiority in fluorescence imaging/sensing, such as near infrared imaging, photostability, biocompatibility and low toxicity. Their characteristics naturally integrate therapy and diagnosis, and when CDs are combined with targeted chemical/genetic/photodynamic/photothermal therapeutic moieties, they are good candidates for targeting TAMs. We concentrate our discussion on the current learn of TAMs and describe recent examples of macrophage modulation based on carbon dot-associated nanoparticles, emphasizing the advantages of their multifunctional platform and their potential for TAMs theranostics.

## Introduction

Overall, 1,918,030 new cancer cases are expected to be diagnosed in the United States in 2022, equivalent to 5,250 new cancer patients per day. Men have a lifetime cancer probability of 40.2%, slightly higher than women (38.5%), and it is also the leading cause of death. In recent years, immunotherapy has emerged as a rising star in the cancer therapeutics spectrum and is a promising strategy for cancer treatment (1). Regrettably, definitive durable therapeutic effects are observed in a small proportion of patients. But the majority show limited clinical benefit or no response at all (2). In order to overcome the resistance of immunotherapy, the mechanism of immunosuppression has been studied in depth in recent years. Numerous studies have shown that the tumor microenvironment (TME) plays an important role in immunosuppression. Multiple inhibitors in the tumor microenvironment (TME) have been identified through analysis. Cell populations, among which tumor-associated macrophages (TAMs) stand out, are promising new targets for tumor immunotherapy (3). TAMs are a prevalent type of inflammatory cell found in the stroma of various tumors. They exhibit a diverse range of phenotypic characteristics and contribute to tumor growth, metastasis, and recurrence by facilitating an immunosuppressive environment. In solid tumors, TAMs are closely associated with poor prognosis.

Despite the complex phenotype, macrophages can be divided into two subtypes based on function: M1 (anti-tumor immunity) and M2 (immunosuppression and tumor immune evasion through suppression of T cell function). M1 macrophages secrete pro-inflammatory cytokines and chemokines, present antigens professionally, participate in positive immune responses, and play a role in immune surveillance. In contrast, M2 macrophages have weaker antigen presentation abilities and primarily inhibit immune responses through their secretions. Cytokines such as IL-10 and TGF-β can down-regulate immune responses, with M2 macrophages as the central players. When combined with other immunosuppressive cells in the tumor microenvironment (TME), these factors not only cannot exert anti-tumor activity but can also create a favorable environment for tumor growth and metastasis. Therefore, evaluating the balance between M1 and M2 macrophages can be a useful strategy for characterizing the immune landscape of the tumor microenvironment. Higher levels of tumor-infiltrating M2 were significantly associated with shorter survival, while higher proportions of M1 with pan-macrophages (%M1) showed a positive correlation with longer overall survival (4). At present, a variety of related small molecule drugs have been developed targeting TAMs (5). Nevertheless, the lack of targeting of these small molecule drugs and the complex microenvironment of solid tumors in clinical trials have limited the efficacy of these small molecule drugs to a certain extent (6). Nanomaterials possess a diverse range of physicochemical properties that enable them to function as both delivery carriers and immunomodulators, making them a promising avenue for improving the immunosuppressive microenvironment of tumors. Research on polarization induced by nanomaterials has focused on a variety of materials, including carbon-based materials, iron oxide nanoparticles, gold particles, zinc oxide particles, and more (7).

As a new member of the family of carbon nanomaterials, CDs are small carbon-based nanoparticles that have gained a lot of attention in recent years due to their unique optical, electrical, and chemical properties, such as small-scale morphology, easily functionalized surface, and tunable optical properties (8). These properties make CDs attractive for a range of applications, including in medicine.

One of the most promising applications of CDs in medicine is in the field of bioimaging (9, 10). CDs can be easily conjugated with biomolecules such as proteins, antibodies, or nucleic acids, and can be used as fluorescent probes to visualize cells, tissues, and organs (11).

Carbon dots offer several advantages compared to traditional organic dyes or semiconductor quantum dots, such as low toxicity, good biocompatibility, and high photostability. They can be used in various bioimaging applications, including fluorescence imaging, intracellular imaging, and biosensors. Carbon dots have a high quantum yield, making them effective fluorescent probes for detecting cancer cells, pathogens, and other biological targets (12, 13). Their small size and ability to penetrate cell membranes make them ideal for imaging intracellular structures and studying cellular processes such as endocytosis, exocytosis, and cell division (14–16). In addition, carbon dots can be used as biosensors to detect specific biomolecules or environmental factors, such as glucose, heavy metals, and other chemicals in biological and environmental samples (17, 18).

Another potential application of CDs in medicine is in drug delivery (19). CDs can be functionalized with different types of molecules such as drugs, peptides, or nucleic acids, and can be used to deliver these molecules to specific cells or tissues. CDs have shown promise for delivering drugs to cancer cells, for example, by targeting tumor-associated macrophages or by enhancing the therapeutic efficacy of chemotherapy drugs (20, 21).

In addition to their potential applications in bioimaging and immunotherapy, carbon dots have also been investigated for their antibacterial and antiviral properties (22–24). These nanoparticles have been found to inhibit the growth of various types of bacteria and viruses, including drug-resistant strains, and have been proposed as a potential alternative to traditional antibiotics or antiviral drugs (25–27).

Overall, the theranostic potential of carbon dots (CDs) and associated nanoparticles is rapidly advancing due to their unique optical properties and versatility in preparation and functionalization. CDs have been utilized for imaging macrophages and tracking their movement in tissues, due to their high quantum yield and photostability (28). Given the intrinsic physicochemical properties and multifunctionality of CDs, their interactions with TAMs offer exciting possibilities that are worth exploring. Currently, there is limited research on carbon dot-targeted TAM imaging, diagnosis, and treatment, although studies have demonstrated their potential in inflammation and antibacterial applications. CDs have also shown promise in immunotherapy, where they can stimulate the immune system to fight diseases. This review paper primarily focuses on analyzing the potential of CD-associated nanoparticles in targeting TAMs, summarizing their application in monitoring and regulating macrophages, and highlighting current challenges in this field.

## Characteristics of tumor-associated macrophages

### Origin, phenotypes, and function of TAMS

Macrophages are distributed throughout body tissues with the functions of phagocytosis and in response to inflammatory signals strategically. Tissue macrophages are derived from embryonic or adult hematopoietic stem cell (HSC) progenitors, and the relative contribution of these cell populations varies from tissue under homeostasis conditions (29). A monocyte is a kind of white blood cell that is made in the marrow and travels through the blood to tissues in the body where it becomes a peripheral monocyte reservoir or non-classical patrolling monocyte or tissue-resident macrophage in the steady state (30). Macrophages respond to the combined stimulation of the origin and resident tissue which contribute the polarization responses (31).

In most human solid malignancies, tumor-associated macrophages (TAMs) and their precursors occupy the most significant portion of bone marrow infiltration, which can account for up to 50% of the total solid tumor volume (32). A large number of current studies show that the localization and density of TAMs are related with poor clinical outcomes in some kinds of solid cancers, including bladder, breast, liver, renal, prostate, and gastric cancer (33–40). Monocyte-derived TAMs take a large part of tissue-resident macrophages in tumors, except for a small part of TAMs derived from tissue-resident macrophages (41). Monocytes are recruited by chemokines (CCL1, CCL2, and CCL5), VEGF, PDGF, TGF-β and CSF -1. Among these cytokines, CCL2 plays a major role in the recruitment of monocytes (42–48). Studies have shown that targeting the CCL2-CCR2 axis could effectively reduce tumor growth and metastasis in mouse models (49). After being recruited to the TME, monocytes can differentiate into M1-like macrophages (pro-inflammatory and usually anti-tumor) and M2-like macrophages (anti-inflammatory and pro-tumor) due to the heterogeneity of the microenvironment (50–52). More and more evidence suggests that TAMs are similar to normal macrophages in their capacity for adopting a broad range of intermediate activation states, reflecting the diverse microenvironmental conditions and rich plasticity according to different signals in the tumor microenvironment (5, 53).

The phenotype of tumor-associated macrophages (TAM) is driven by both the tumor microenvironment (TME) and the tumor immune microenvironment (TIME). Under the influence of TIME, adaptive and innate immune cells provide chemical messengers for regulating the functional phenotype of macrophages, such as immunoglobulin secreted by B cells, IL4 and IL13 secreted by TH2 cells, Treg cells secreted IL10 and TGFβ, as well as IFNγ and TNF secreted by NK cells, CTL and TH1 cells. In the TME, cytokines secreted by tumor cells, tumor-associated fibroblasts, directly affect the phenotype of TAMs, while oxygen deficiency, fibrosis, and cellular stress also customize the phenotype of TAMs. Thus, immune-related and non-immune-related factors jointly drive functional or dysfunctional antitumor immune. TAMs are programmed to drive inflammation when the microenvironment has functional vasculature, normoxia, low extracellular matrix density, more TH1 cells than TH2 cells, and high cytotoxic T cell (CTL) infiltration. Macrophages exhibit a robust antitumor adaptive immune response. In contrast, tumor hypoxia and fibrosis are combined with infiltration of large amounts of cancer-associated fibroblasts (CAFs) and immunosuppressive cells, and macrophages are programmed to promote a pro-tumor phenotype of immunosuppression and tissue remodeling, resulting in cytotoxicity T lymphocyte (CTL) rejection and suppression.

Studies have shown that M1 phenotype macrophage is stimulated by cytokines such as IL12, TNF, and IFNγ, microbe-associated molecular patterns (MAMPs) such as bacterial lipopolysaccharide (LPS), or other Toll-like receptors (TLR) agonists (54–57). In contrast, anti-inflammatory M2 macrophages are polarized by the stimulation of some of cytokines such as IL4, IL5, IL10, IL13, CSF1, TFGβ1 and PGE2 (58, 59).

M1-type TAMs can express factors such as nitric oxide synthase(iNOS), reactive oxygen species (ROS), and IL-12 that have the functions of phagocytosis and killing target cells (60). M2-type TAMs are associated with high expression of IL-10, IL-1β and VEGF *in vivo*. They can also express a large amount of scavenger receptors, which have the functions of clearing debris, promoting angiogenesis, tissue reconstruction, and injury repair and promote the function of tumorigenesis and development (61–63). Patients with more M2 TAMs infiltration have a lower survival rate and an increased lymph node metastasis rate (64). In general, both M1 and M2 TAMs exhibit strong intrinsic plasticity, can cross-regulate each other’s functions, and do not represent a fixed, frozen phenotype; M1 and M2 TAMs can co-exist in the same tumor microenvironment; therefore, molecular targets that control polarization balance may be important avenues for tumor immunotherapy. Polarization biomarkers for M1-type macrophages include CD86 and CD80, and for M2-like macrophages include CD163, CD204, CD206, CD115, and CD301 (65).

The induction of monocytes into the tumor microenvironment into M1/M2 macrophages also changes dynamically with the development of tumors. In the early stages, macrophages can recognize and present malignant cells to lymphocytes. Early stages of tumors exhibit a limited degree of hypoxia, at which time the immune microenvironment exhibits an immunostimulatory state, such as a massive infiltration of effector T cells and polarization of tumor-associated macrophages (TAMs) to an M1-like state (66). As the tumor progresses, cancer cells consume a large amount of glucose. They produce more lactate, which promotes the generation of a hypoxic environment, and the secretion of cytokines also facilitates the recruitment of hematogenous monocytes. It promotes them to an immunosuppressive M2-like state of polarization (67).

**Scheme 1:**
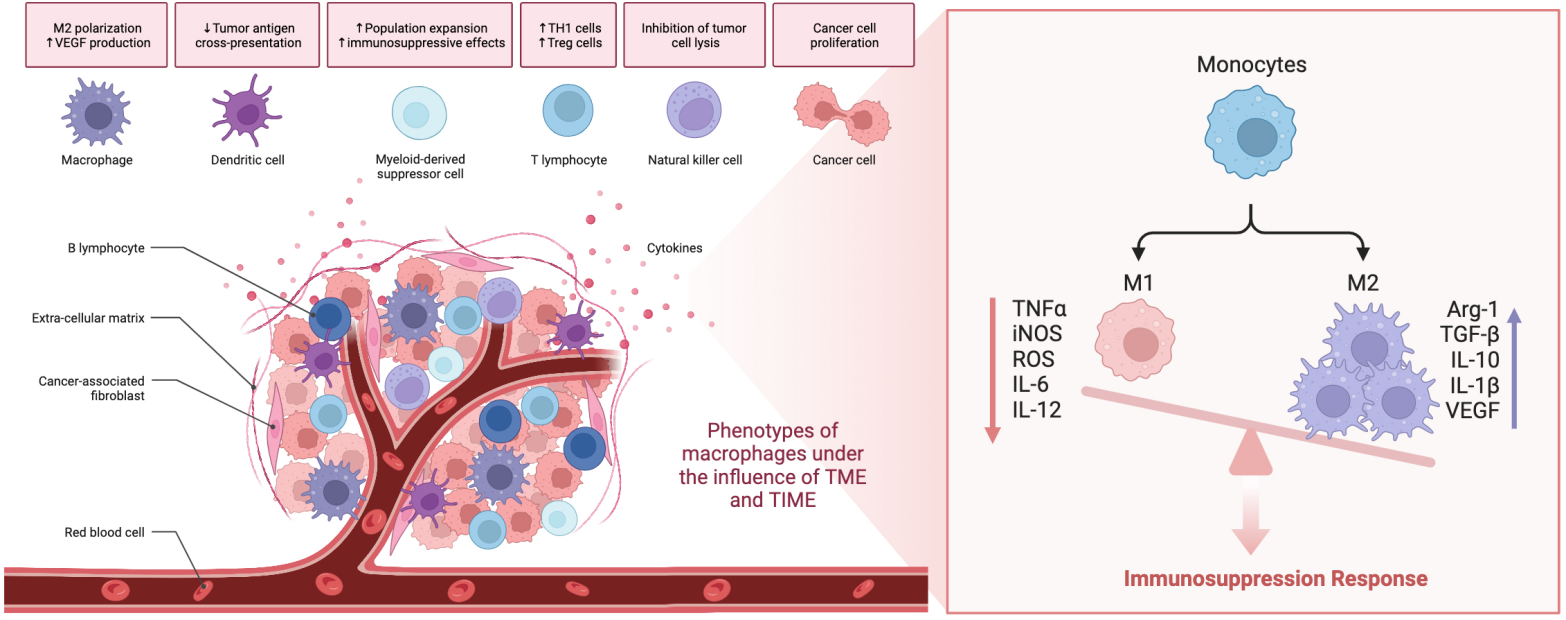
The role of TAMs in the tumor microenvironment.

TAMs are the center of inhibiting the ability of T cells in tumors to respond, and the current limitations of various immunotherapies are closely related to this, especially those related to immune checkpoints. One study showed that TAM and CD8+ T cells engage in specific, persistent, antigen-specific synaptic interactions that not only fail to activate T cells but actually exhaust them and accelerate the process under hypoxic conditions (68). The current findings indicate that TAMs can regulate T cells through direct and indirect pathways, respectively (69). Tumor-associated macrophages (TAMs) can directly inhibit cytotoxic T cells through three pathways. Macrophages are involved in immunosuppression through a variety of mechanisms, such as expressing immune checkpoint molecules, including programmed cell death 1 ligand 1 (PDL1) (51), producing inhibitory cytokines like IL-10 and transforming growth factor-β (TGF-β) (52), and modulating their metabolic activity by consuming metabolites (such as L-arginine) and producing reactive oxygen species (ROS) (70, 71). In this summary, we highlight the effects of immune cells and the alterations in macrophage phenotype that occur within the tumor microenvironment and immune microenvironment (**Scheme 1**).

### Modulating TAMs for tumor immunotherapy

**Scheme 2:**
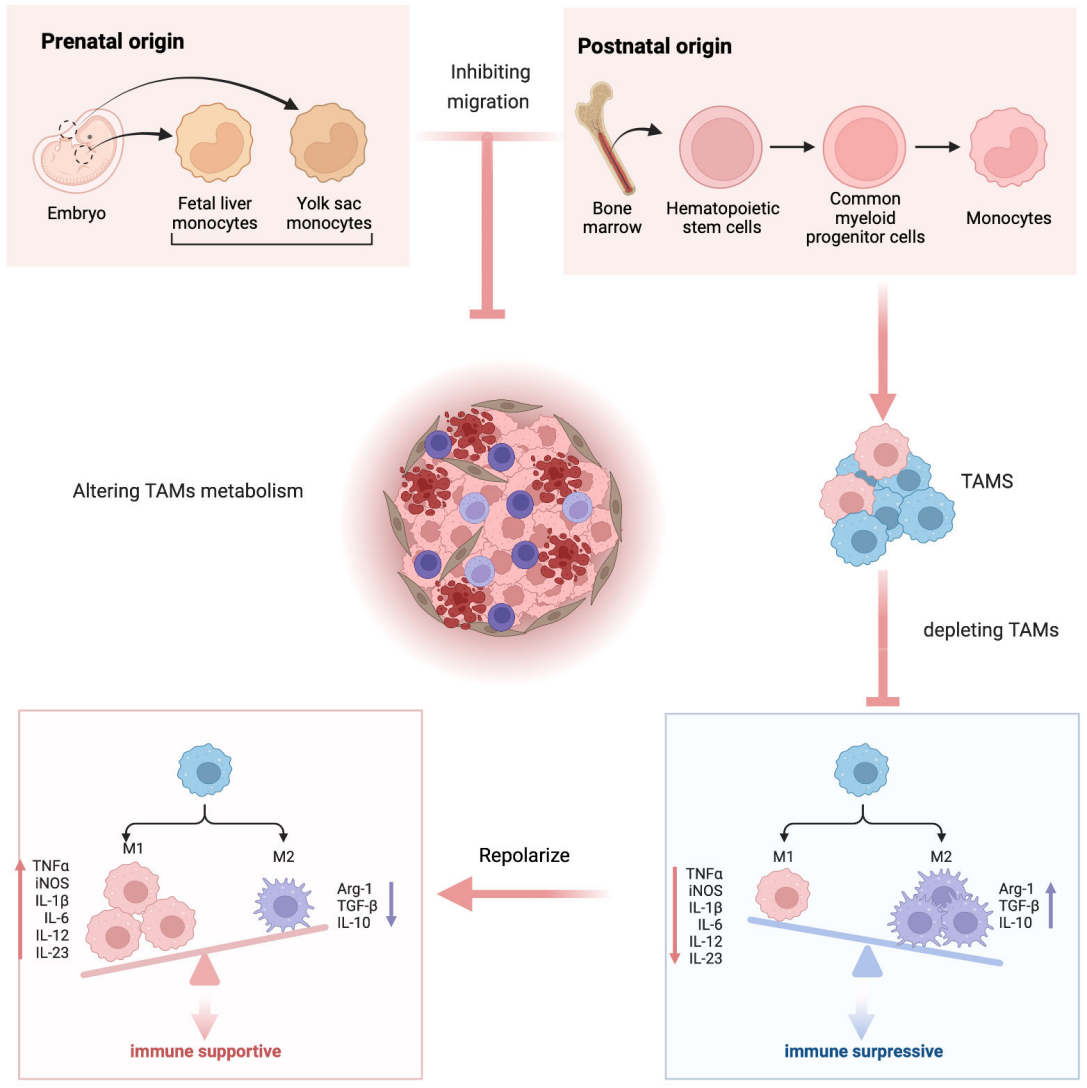
Schematic representation of modulating tumor-associated macrophages as immunotherapy.

Depending on the different sources and phenotypes of TAMs, tumor immunotherapy targeting macrophages can be divided into four categories (1): inhibiting the migration of monocytes or M-MDSCs to tumors (2), depleting TAMs (3), repolarizes TAMs (4), altering TAM metabolism (72), as shown in (**Scheme 2**). Since conventional modulators of TAMs face challenges such as non-specific targeting, limited drug delivery efficiency, rapid blood clearance, and systemic toxicity, nanoparticles are rationally designed to deliver them or directly participate in regulation, as they can be designed with tunable dimension and surface charge, Moreover, nanoparticles can be easily internalized by the phagocytosis inherent in macrophages, which promotes the effective accumulation of nanoparticles and their payloads in tumors to enhance their tumor penetration (73). Therefore, engineered nanoparticles for targeted delivery of TAMs to tumors or direct modulation of TAMs have enormous potential to strengthen tumor-specific accumulation and modulator blood circulation time and thus reduce side effects, which can enhance TAMs modulatory efficacy (7). Here, we will analyze whether CDs-associated nanoparticles can regulate the possibility of TAMs based on the above four strategies.

## Mechanism and importance of carbon materials in TAMs

The remarkable physicochemical properties and biocompatibility of carbon-based materials have sparked significant interest in their potential applications in cancer immunotherapy. These nanomaterials exhibit distinctive characteristics that make them highly promising for biomedical imaging and therapy. They have been extensively investigated for their ability to facilitate one-photon and two-photon imaging, which makes them ideal for both shallow and deep-tissue imaging. Additionally, their ease of functionalization and biocompatibility allow for targeted delivery of therapeutic agents and imaging agents. As research progresses, carbon-based nanomaterials have the potential to become valuable tools in the diagnosis and treatment of a range of diseases, including cancer (74–78). Rajendra K. Singh and Hae-Won Kim and their team have developed a novel type of nanoparticles called fluorescent mesoporous bioglass nanoparticles (fBGn) that can be used for cancer diagnosis and treatment. These nanoparticles are based on carbon dots (CD) and possess a variety of beneficial properties, including triple-mode imaging, photodynamic and photothermal therapeutic effects, and the ability to deliver anticancer drugs in a pH-dependent manner. The researchers were able to demonstrate the effectiveness and biocompatibility of fBGn *in vivo* using a nude mouse model. The authors suggest that fBGn hold great promise for cancer theranostics due to their multifunctional capabilities for imaging, drug delivery, and therapy (79). Singh and Kim have developed a novel nanoplatform called C-dot bioactive organosilica nanosphere (C-BON) that has the potential for therapeutic and diagnostic purposes in tissue repair and disease treatment. This platform has several advantages, including its ability to label cells and tissues, load and deliver drug molecules, and exhibit photothermal activity. Additionally, the C-BON has demonstrated excellent bioactivity and cell compatibility, making it a promising candidate for future applications in theranostics. Overall, this innovative technology offers a multifunctional approach to chemotherapy and photothermal therapy with optical imaging, paving the way for improved treatments in the future (80).

Carbon-based materials, such as carbon nanotubes, graphene oxide, and fullerenes, have been shown to modulate TAMs’ activation state and promote an anti-tumor immune response (78, 81, 82). One of the mechanisms by which carbon-based materials can achieve this is through the regulation of TAMs’ phagocytic activity. Carbon-based materials have been shown to enhance TAMs’ phagocytosis of tumor cells, leading to their subsequent destruction and increased activation of the immune system against the tumor (83).

In addition, carbon-based materials can also promote the polarization of TAMs towards an M1-like phenotype, which is associated with an anti-tumor immune response (82). This is achieved through the activation of toll-like receptors (TLRs), which are involved in the recognition of pathogen-associated molecular patterns (PAMPs) and damage-associated molecular patterns (DAMPs) on the surface of cancer cells (77, 84, 85).

Moreover, carbon-based materials can also act as a drug delivery platform for targeted delivery of anti-cancer agents to TAMs (86). This targeted delivery can increase the efficacy of anti-cancer agents and minimize their off-target effects.

Overall, carbon-based materials have emerged as a promising strategy for modulating TAMs’ activation state and promoting an anti-tumor immune response. The unique properties of carbon-based materials make them an attractive candidate for further development in cancer immunotherapy.

## Characteristics of CDs

Carbon-based nanostructured substances, such as graphene, carbon nanotubes and fullerenes, have attracted wide attention due to their unique physical and chemical properties and diverse applications. Compared to the above carbon nanostructures, carbon dots (CDs) exhibit excellent dispersion, low toxicity, biocompatibility, biodegradation, abundant raw materials, low cost, and abundant photoluminescence (PL) and photoelectrochemical properties (8, 87). Historically, In 2004 CDs was discovered in arc emission carbon soot, whose PL emission attracted the attention of researchers (88). In 2006, polymers (i.e., PEG, etc.) were used for surface passivation to enhance the PL emission of CDs (89). In 2010, well-crystallized CDs were synthesized and purified, showing size-dependent photoluminescence (90). In general, CDs can be thought of as spherical carbon particles (graphitic fragments) less than 10 nm in size (91). The chemical structure of carbon dots can be a hybrid carbon structure of sp2 and sp3, with a single-layer or multi-layer graphite structure, or it can be aggregated particles of polymers. Specifically, carbon dots include graphene quantum dots (GQDs), carbon quantum (CQDs), and polymer dots (CPDs). GQDs refer to a carbon core structure with a single layer or less than 5 layers of graphene and chemical groups bonded to the edges. The size of graphene quantum dots has a typical anisotropy and carbon lattice structure, and the lateral dimension is larger than the vertical height; CQDs are spherical and have a clear lattice, and the surface has abundant chemical groups CQDs have an intrinsic state luminescence mechanism and a quantum confinement effect of particle size. CPDs are usually cross-linked flexible aggregates formed from non-conjugated polymers through dehydration and partial carbonization, and there is no carbon lattice structure. Currently, four fluorescence mechanisms have been reported as follows (1) quantum confinement effect (QCE) (2), defect state (3), molecular (fluorophore) state, and (4) crosslink-enhanced emission state (92). The characteristics of carbon dots have attracted widespread attention in the field of biomedicine. Currently, CPDs are the core of research and development of carbon dot materials. The excellent properties of CPDs, such as photostability, excellent biocompatibility, simple synthetic route, flexible designability, deep red/NIR emission, and two-photon/multiphoton fluorescence, make CPDs an ideal candidate for fluorescent probes for *in vitro* and *in vivo* bioimaging (19, 93).

## Synthesis strategy of carbon dots for bioimaging and therapy

There are many methods for preparing carbon dots, which can be generally divided into top-down method (Top-down) and bottom-up method (Bottom-up). The top-down synthesis method is mainly to thoroughly pulverize the carbon skeleton to generate CDs, while the bottom-up method uses some organic molecules as precursors (carbon sources) to synthesize CDs (94). In the history of carbon dots, the top-down strategy was first used to prepare carbon dots, which refers to the synthesis of carbon dots by physically or chemically stripping carbon nanoparticles from large carbon skeletons, including discharge methods, electrochemical methods, etc. method, laser ablation method, etc. (95, 96). Although these methods can generate CDs in relatively large quantities, they often suffer from expensive instrumentation, complex synthesis procedures, long synthesis times, low yields, high impurities, complex purification procedures, and still require post-synthesis procedures to tune optoelectronic properties (97). From the perspective of fluorescence properties of CDs, the oxidative cleavage of carbon sources leads to more structural defects, which leads to the degradation of photoluminescence performance, which is the most restrictive issue for their biomedical applications (98).

Bottom-up synthesis is more prevalent now (99). The advantage of this strategy is the availability of a large number of molecular precursors, among other benefits including multiple heat treatment options, faster reaction times and more uniform properties of the final material. The selection of precursors and synthesis procedure (i.e., pre-synthesis control) affects the physicochemical properties of CDs in terms of size, degree of graphitization, surface functional groups, and doping. However, some structural and functional features of the precursors can be retained in the nanoparticles, which allows a certain degree of predictability in the designed nanoparticles. At the same time, the strategy of using heteroatom doping can enrich the functional properties of carbon dots and adjust the range of photoluminescence.

Bottom-up synthetic strategies can obtain nanoparticles emitting from the blue to the near-infrared (NIR) region (100). The bottom-up method mainly uses some organic molecules as precursors to prepare CDs through a series of chemical reactions, including template method, microwave digestion synthesis method, ultrasonic oscillation method, solvothermal method, strong acid oxidation method and hydrothermal method, etc. Among these methods, hydrothermal method, solvothermal method and template method are widely used (101). CDs can be functionalized by surface passivation and heteroatom doping (102). With proper functionalization, carbon dots have promising applications in biomedical fields such as biosensors, bioimaging, and photodynamic therapy; magnetic resonance imaging of chemical exchange saturation transfer; photodynamic and photothermal therapy; PH and ROS in microenvironments monitor and treatment (103–109).

## Application of carbon dot-associated nanoparticles in monitoring macrophages

Currently, cancer treatment response is routinely assessed with the Response Evaluation Criteria in Solid Tumors (RECIST), based on changes in tumor size and the presence or absence of new tumors (110). However, in immunotherapy, pseudoprogression has emerged as a distinct response mode in which activated immune cells infiltrate the tumor environment leading to increased tumor volume and delayed treatment response (111). Because TAMs are the highest proportion of immune-infiltrating cells in tumors and their substantial impact on immunotherapy, immunoimaging of TAMs is essential to evaluate changes in tumor burden, allow early treatment intervention, reflect the dynamic shift in immune markers during immunotherapy, and avoid early termination of effective therapy according to RECIST criteria (112).

Thanks to carbon dots’ inherent fluorescence characteristics and physical and chemical properties, it has the intrinsic advantage of being a macrophage imaging agent. Raja S and co-workers synthesized a carbon dot derived from curauá that exhibited a graphitic-like structure with an average diameter of 2.4 nm, good water solubility, sophisticated carboxyl and hydroxyl functional groups, excitation-dependent multicolor fluorescence emission (in the range of 450 nm to 560 nm) and excellent photostability. Cell experiments show that carbon dots tolerate the J774.A1 mouse macrophage cell line, can effectively internalize carbon dots into its cytoplasmic compartment and is an excellent nanoprobe for effective long-range cell imaging (113).

Xiaowei Xu and colleagues aimed to develop a carbon nanoparticle incorporating aspirin. They synthesized fluorescent aspirin-based carbon dots (FACD) through a one-step microwave-assisted method, condensing aspirin and hydrazine. Imaging data revealed that FACD effectively penetrated mouse monocyte-macrophage cells *in vitro* (114).

Shi Y et al. synthesized highly fluorescent and ultra biocompatible N-doped carbon quantum dots derived from aminated alkali lignin green precursors for cellular imaging and intracellular irons detection of RAW 264.7 cells. AL-CQDs produced in the 4–10 nm range exhibited excitation-dependent and pH-stable fluorescence properties. They were used to detect iron ions ranging from 100 nm to 1 mm with a detection limit as low as 8 nm, where Fe3+ ions could be detected by the AL-CQDs. The amine group is trapped, forming an absorbing complex that results in significant fluorescence quenching (115).

Yawei Li and colleagues fabricated stable nanoparticles composed of the supramolecular assembly of carbon dots (CDs) and RTBs, which could be taken up and visualized by macrophages. Notably, the CDs-RTB nanoparticles were found to promote macrophage proliferation, as well as the production of NO, IL-6, and TNF-α in RAW264.7 cells, and increase mRNA expression, indicating enhanced immunomodulatory activity. These findings highlight the potential of CDs as a simple and stable platform for assembling RTB, thereby facilitating the application of RTB as an immunostimulant (116).

The photoluminescent properties, low toxicity, and biocompatibility characteristics of these carbon dots exhibit excellent properties in bioanalysis and bioimaging. However, fabricating stable highly near-infrared (NIR) fluorescent GQDs using facile methods remains a challenging task. Reagen S and a co-worker developed NIR CDs from the biomass-derived organic molecule cis-cyclobutane-1,2-dicarboxylic acid *via* one-step pyrolysis. The prepared GQDs exhibit excellent photostability and stability over a wide pH range. Using biomass as raw material to prepare carbon dots is a very convenient and economical method. Most importantly, there were two peaks in the fluorescence emission spectra of GQDs, one in the NIR region around 860 nm. The results of cell experiments on the mouse macrophage cell line RAW 246.7 showed that GQDs entered cells by endocytosis on fluorescence images and were nontoxic to cells at concentrations up to 200 μg/mL (117).

At the same time, the CDs-based composite material has more prosperous functions, which can significantly improve CDs’ cellular uptake and imaging potential. It was shown that nanocomposite formulations of carbon dots (<5 nm) encapsulated in lipid-based lyotropic liquid crystal nanoparticles (~250 nm) enhanced the bioimaging potential of carbon dots by improving cellular uptake efficiency and converging carbon dot light emission (118). Carbon dot-associated nanoparticles enable multimodal imaging by doping with heteroatoms or forming assemblies. Sun S and co-workers anchored a small amount of photosensitizer chlorin e6 (Ce6) (0.56% by mass) on amino-rich red-emitting carbon dots (RCD). They synthesized Ce6-modified RCD (named Ce6-RCD) multimodal imaging capability (i.e., fluorescence (FL), photoacoustic (PA), and PT) (119).

Saladino GM et al. synthesized metallic rhodium (Rh) nanoparticles conjugated and cross-linked with nitrogen-doped carbon quantum dots, which combine optical and X-ray fluorescence as multimodal bioimaging contrast agents. CQDs confer optically fluorescent properties to Rh NPs and improve their biocompatibility, as demonstrated *in vitro* by real-time cell analysis (RTCA) on a macrophage cell line (RAW 264.7) (120).

Su Y and colleagues developed Hafnium-doped carbon dots (HfCDs) using a simple one-pot pyrolysis method. This innovative nanoparticle exhibited remarkable capabilities for CT/fluorescence imaging (9).

By doping Gd (iii) into CQDs *via* one-pot pyrolysis, Pan Y et al. reported an efficient and mild method for the facile synthesis of carbon quantum dots (CQDs)-based bimodal fluorescent (FL)/Magnetic resonance (MR) imaging probe cryogenic process. Nanoparticles doped with heavy N elements can significantly improve the quantum yield. Gd3+ is stably captured and sequestered by the carbon dot framework, maximizing its role in shortening the longitudinal relaxation time. Therefore, the synthesized nanoparticles have the advantages of strong fluorescence brightness and high MR response with minimal Gd3+ extravasation, making them an ideal dual-modality imaging probe (121).

He X et al. prepared novel carbon dots (CDs) L-CD/C-CD from Gd (iii) salt/complexes, cationic polymers, and citric acid, which combine the abilities of gene delivery and multi-modal (MR/FL) imaging (122).

Weng Y and co-workers et al. report a multifunctional nanocarrier (CDs/ICG-uLDHs) prepared by simple self-assembly of red-emitting carbon point (CDs) and indocyanine green (ICG), which can be used for three-mode fluorescence/photoacoustic/two-photon bioimaging and high-efficiency photothermal therapy (123). By doping rare earth ions, carbon dot composites can obtain excellent UCL imaging, magnetic resonance imaging (MRI), and computed tomography (CT) imaging performance (124).

The multifunctional hybrid nanoparticles prepared by Wang H et al. have fluorescence/MRI dual-mode imaging capabilities, which are made by embedding a magnetic Fe3O4 core into a mesoporous silica shell of carbon point (CD) and paclitaxel (PTX), covered by another layer of silica (21).

In addition to direct cell imaging, carbon dots also serve as sensitive sensors to rapidly image reactive oxygen species (ROS) and reactive nitrogen species (RNS) signals involved in various biological processes and many pathologies with high selectivity and contrast. Gong Y et al. developed phosphorus and nitrogen co-doped carbon dots (PC-NDs). ROS and RNS can sensitively and selectively quench the strong fluorescence of PN-CD *in vitro* and *in vivo*. It can be used for live-cell imaging of reactive oxygen species (ROS) and reactive nitrogen species (RNS) in macrophages. The carbon dots prepared by Yu C et al. are highly selective for NO and can be operated in an utterly aqueous medium, which can track exogenous NO levels in various cell lines such as Raw 264.7, L929 and Hela cells; it is also used to visualize endogenously produced NO stories in the Raw 264.7 macrophage cell line (125).

**Figure 1:**
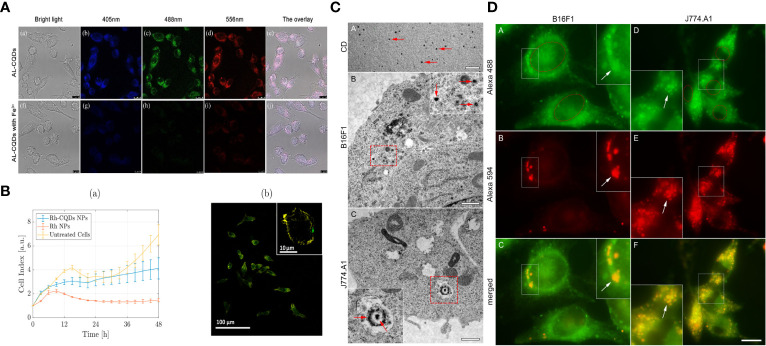
Bioimaging of macrophages with various CDs :**(A)** Fluorescence images indicated AL-CQDs could detect iron ions of RAW 264.7 cells. **(B)** The RTCA assay demonstrated that Rh-CQDs NPs can enable multimodal imaging in the RAW 264.7 cell line. **(C, D)** Transmission electron microscopy and Fluorescence microscopy analysis shows that C-dots can be stably imaged in B16F1 and J774.A1 cytoplasm.

Studies as shown in **Figure 1** illustrate that different protocols for multimodal imaging in monitoring macrophages can be achieved with appropriate surface functionalization, heteroatom doping, and assembly of carbon dot-associated nanoparticles (**Figure 1**).

## Application of carbon dot-associated nanoparticles in regulating macrophages

CDs are generally soft and nontoxic *in vitro* and *in vivo*. However, due to their efficient light harvesting in an extensive spectral range from ultraviolet to near-infrared, CDs exhibit strong photodynamic effects, and photoexcited CDs can generate reactive oxygen species (ROS). ROS is a crucial mediator of oxidative stress and redox signal transduction in immune cells (126–128). The regulation of ROS by CDs may have a profound impact on the immune response. Yu Jin et al. found that CDs can reprogram macrophages by eliminating ROS to suppress pro-inflammatory responses and promote pro-reparative M2 conversion (129). Huibo Wang and colleagues found that carbon dots (CDs) produced through a one-step hydrothermal process using citric acid and glutathione exhibited excellent intracellular reactive oxygen species (ROS) scavenging activity in macrophages. This scavenging activity was effective in reducing inflammation caused by lipopolysaccharide (LPS) induction in macrophages, suggesting that CDs have potential as a therapeutic agent for inflammatory conditions. Studies have found that CDs can effectively remove up to 98% of intracellular ROS, especially inhibit the nuclear factor kappa-light chain enhancer (NF-κB) signaling pathway of activated B cells, reduce the expression level of inflammatory factor IL-12, thereby regulating Macrophage phenotype (130). At the same time, many studies have shown that carbon dots can induce autophagy (131–133). Mitochondrial ROS plays a key role in promoting macrophage polarization into an inflammatory phenotype by damaging the autophagolysosome system (134). Therefore, carbon dots may regulate immune responses through these two aspects and impact on macrophages (135–137). A study shows that degradable carbon dots (CDs-1) prepared from L-ascorbic acid can up-regulate the expression of HMOX1 in animal cells and tissues, and can increase the expression of HMOX1 by 5 times in a short period of time, thereby reducing cell inflammation ROS levels in models with therapeutic effects on LPS-induced acute lung injury in mice (138).

In another study, researchers synthesized highly biocompatible CDs (Gly-CDs) by hydrothermal method using glycyrrhizic acid, an active ingredient of Chinese herbal medicine, as a raw material. The results indicated that Gly-CDs inhibited the invasion and replication of PRRSV, stimulated the antiviral innate immune response, and inhibited the accumulation of intracellular reactive oxygen species (ROS) caused by PRRSV infection (139).

Osteoclasts, specialized cells derived from the fusion of monocyte/macrophage hematopoietic lineage precursors, are the primary cells involved in normal bone remodeling and pathological bone destruction *in vivo*. One of the main causes of hyperactivation of osteoclasts is the overproduction of reactive oxygen species. Chitosan-derived nitrogen-doped carbon dots (N-CDs) synthesized by Chen Runfeng et al. have the ability to scavenge reactive oxygen species (ROS). Experiments showed that N-CD effectively abolished the RANKL-induced increase in ROS generation, thereby attenuating the activation of NF-κB and MAPK pathways, whereby osteoclast genesis and bone resorption were effectively attenuated *in vitro*. Furthermore, N-CD protected mice from lipopolysaccharide (LPS)-induced calvarial destruction and breast cancer cell-induced tibial bone loss. Based on the excellent biocompatibility and efficient ROS scavenging ability of N-CDs, for the first time, it provides a nanomaterial treatment plan for the clinical treatment of osteolytic diseases (140).

Cai H et al. synthesized a carbon dot capable of simultaneously achieving cell labeling and regulating mesenchymal stem cell (MSC) behavior. Bifunction CDs were prepared with D-glucosamine hydrochloride and sodium p-styrene sulfonate as raw materials by one pot hydrothermal method. The synthesized CDs had uniform particle size (about 4 nm), was well dispersed in aqueous solution, and showed excellent fluorescence stability under other conditions. More importantly, CDs can effectively promote osteogenic and chondrogenic differentiation of rBMSCs through the production of reactive oxygen species (ROS), without affecting their pluripotency (141). Shao D et al. also had similar results with citrate-based carbon dots, which significantly provided long-term tracking and promoted the differentiation of rBMSCs into osteoblasts through the ROS-mediated MAPK pathway (142).

Injection of GQDs was able to penetrate the blood-brain barrier, inhibited the loss of cerebellar Purkinje cells, and demonstrated reduced microglial activation. Microglia are macrophages in the brain, suggesting that carbon dots can regulate macrophages through autophagy (143). Another study shows that electrochemically produced CDs irradiated with blue light (470 nm, 1W) produce reactive oxygen species, including singlet oxygen. Light-excited CD-induced cell death is manifested by apoptosis (externalization of phosphatidylserine, activation of caspases, DNA fragmentation) and autophagy (autophagy vesicles formation, LC3-I/LC3-II transformation, morphological and/or biochemical characterization of autophagy target p62) (144).

The results of Yiru Qin et al. revealed that CDs slightly affected the cell viability and membrane integrity of macrophages, while CDs significantly increased reactive oxygen species (ROS) production as well as apoptotic and autophagic cell death, while Bax, Bad, caspase 3, caspase 9 increased expression levels of beclin 1 and LC3-I/II and decreased Bcl-2. In addition, low concentrations of CDs significantly increased the expression of tumor necrosis factor-α (TNF-α), interleukin-1β (IL-1β), IL-8. In contrast, high concentrations of CDs had a negative effect on cytokine production opposite effect. SB202190 is a selective inhibitor of p38 mitogen-activated protein kinase (MAPK), which abolishes the cytokine induction of CD in macrophages. Furthermore, CDs significantly increased the phosphorylation of p38 MAPK and p65 and promoted the nuclear translocation of nuclear factor-κB (NF-κB). These results suggest that CDs induce ROS production, apoptosis, autophagy, and inflammatory responses in THP-1-activated macrophages through p38MAPK and NF-κB-mediated signaling pathways. This indicates that carbon dots have the function of regulating stimulatory factors in macrophages (145).

Carbon dots also offer enormous potential due to their enzymatic properties compared to natural enzymes. Yao L et al. report a carbon dot-based nanozyme prepared from chlorogenic acid (ChA), a primary bioactive natural product in coffee. The study found that ChA CDs exhibited significant GSH oxidase-like activity, which recruited a large number of tumor-infiltrating immune cells, including T cells, NK cells, and macrophages, thereby transforming “cold” tumors into “hot” tumors, activating systemic anti-tumor immune response (146).

Although ricin-binding subunit B (RTB) can promote the activation of macrophages and regulate cell-mediated immunity, its application is severely limited due to the inherent properties of the protein, such as poor stability and low cellular uptake efficiency. In the work of Li Y et al., stable nanoparticles were prepared by supramolecular assembly of carbon dots (CDs) and RTBs. The formed CDs-RTB are highly durable and can protect RTB from enzymatic hydrolysis. More importantly, CDs-RTB could promote the proliferation of macrophages, increase the production of NO, IL-6, and TNF-α in RAW264.7 cells, and increase the expression of mRNA, indicating that CDs-RTB enhanced the immunomodulatory activity. This work highlights the potential of CD as a simple and stable assembly platform that effectively facilitates the application of RTB as an immunostimulatory agent (147). At the same time, it suggested that CD has the potential to be an excellent immune adjuvant.

Sun Q et al. have developed a novel nanocomposite to target activated macrophages in the colon with real-time imaging and therapeutic capabilities. The nanocomposite was formed by covalent conjugating mannosylated NPs (Man-NPs) with carbon dots (CDs). Cellular experiments showed greater uptake of nanocomposites by inflamed macrophages compared to untreated macrophages and the mannose receptor-negative cell line 4T1. This indicates that carbon dots can target and recognize M2 macrophages after functionalization (148).

**Figure 2:**
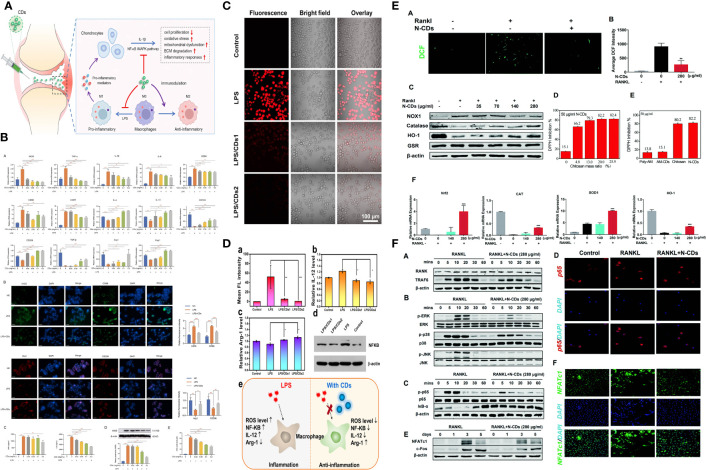
Regulation ROS and autophage with CDs. **(A, B)** CDs orchestrated macrophage repolarization in vitro and indicating the immunomodulatory mechanism of CDs mediated OA therapy. **(C, D)** CDs with radical-scavenging activity in alleviating the LPS-induced inflammation in macrophages. **(E, F)** N-CDs downregulated ROS with suppressed ROS downsteam signaling pathway.

The above studies indicate that carbon dots have the ability to influence macrophage plasticity through several mechanisms. Firstly, they can induce ROS production and autophagy, which can alter macrophage phenotype from M2 to M1-like, resulting in an enhanced immune response against tumors. Secondly, carbon dots can modulate macrophage polarization by inhibiting the expression of cytokines such as IL-10 and TGF-β, leading to an increase in the M1/M2 ratio and improving the characterization of the tumor immune microenvironment. Additionally, carbon dots can act as immunomodulators and delivery vehicles, improving the uptake of therapeutic agents by macrophages and potentially improving the immunosuppressive microenvironment of tumors. These findings suggest that carbon dots may hold promise as a therapeutic approach for targeting TAMs. A relevant mechanism is illustrated in **Figure 2**.

## Concluding remarks and future perspectives

**Scheme 3:**
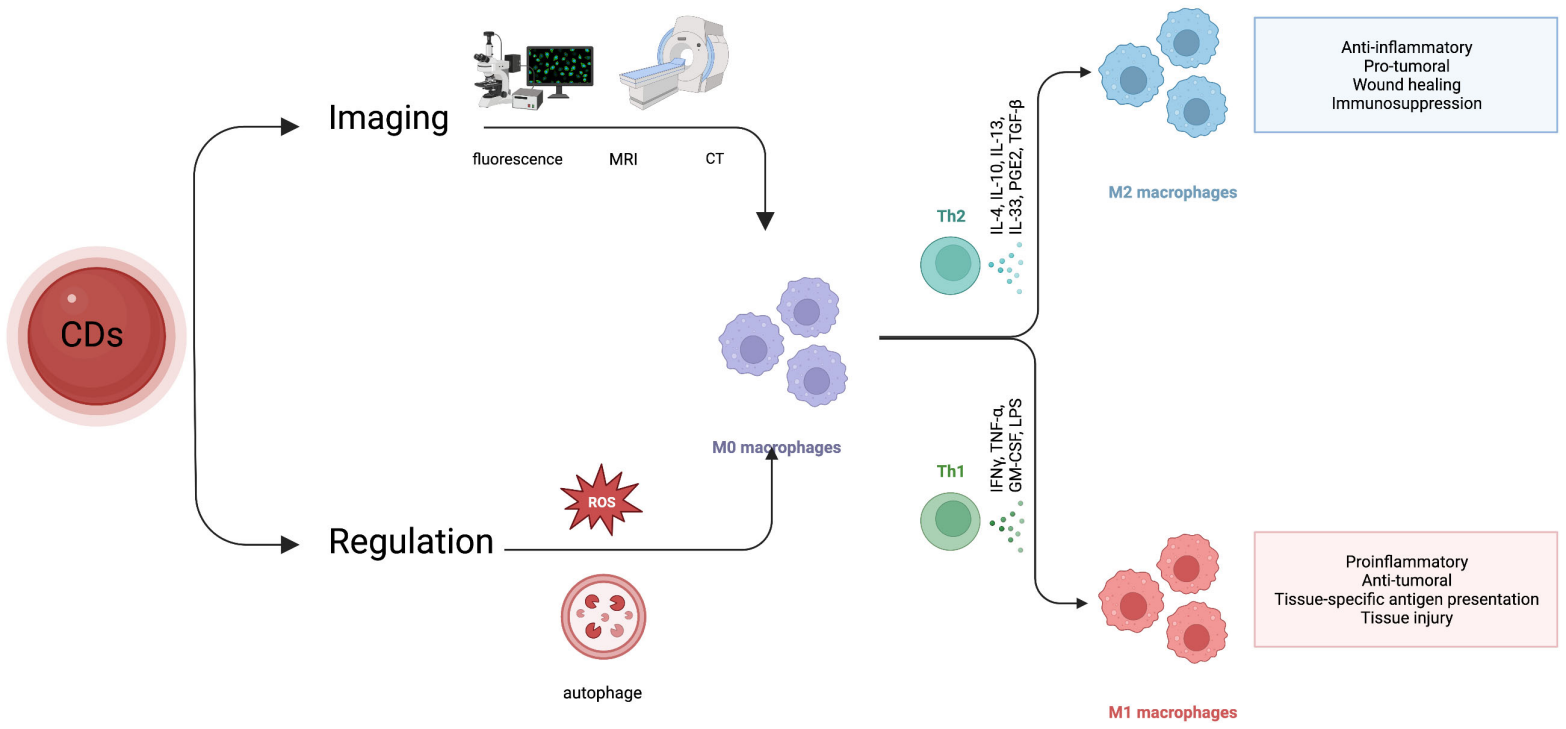
Application of carbon dots in macrophage imaging and modulation.

TAMs contribute to tumor initiation, progression, and metastasis. Therapeutic agents that eliminate TAMs, inhibit TAM infiltration, and/or activate TAM polarization toward the M1 phenotype have shown remarkable clinical potential. Considering the critical role of TAMs in tumor immune suppression, various macrophage-targeting nano theranostics formulations have been developed in recent years. As a new type of nanomaterial, CDs have evolved from a single functional capability of diagnosis (or treatment) in nanomedicine theranostics by their inherent photoluminescence characteristics, excellent physical and chemical properties, and rich tunability. It is an intelligent treatment and diagnosis system. So far, there are few studies on the application of carbon dots in evaluating and regulating TAMs. However, through literature analysis, this review found that CDs have apparent advantages in the imaging and regulation of macrophages. Here, we illustrate the potential of carbon dots in macrophage imaging and regulation (**Scheme 3**). The fluorescence visible in the whole range provides a basis for monitoring macrophage distribution, polarization state, and functional changes. At the same time, the carbon dots exhibited the role of nanozyme and immune adjuvant, which can regulate the polarization state of macrophages and promote the infiltration of immune cells through the ROS generated by photoluminescence and the induction of autophagy. In addition, TAMs are highly enriched in tumor hypoxic sites. This shows that CDs have inherent advantages and great potential for monitoring and regulating TAMs. However, compared with other nanomaterials that have been applied for a long time, the application of CDs in diagnosis and therapy needs to solve more difficulties.

First, further theoretical breakthroughs are required to fine-tune the properties of carbon dots. On this basis, the demand for near-infrared photoluminescence can be stably realized. Second, the tumor microenvironment is complex, and how to achieve safe and efficient target recognition of TAMs is a crucial point that needs to be studied. Third, the current application of carbon dots in macrophages shows a bidirectional effect of ROS and autophagy. Therefore, how to correctly evaluate the state of TAMs and change the immunosuppressive effect of TAMs is very important in the future. Developing multimodal CDs with synergistic strategies may be feasible to achieve this maximal theranostic purpose.

Therefore, with nanomedicine development, CDs are a suitable carrier and a promising reagent for nanomedicine theranostics. If scientists and engineers adequately resolve the above problems, CDs are expected to make outstanding contributions to the development of immunotherapy.

## Author contributions

YM: preparation, creation, and presentation of the published work, specifically writing the initial draft (including substantive translation). BZ: preparation, creation and/or presentation of the published work by those from the original research group, specifically critical review, commentary or revision – including pre-or post-publication stages. LL: acquisition of the financial support for the project leading to this publication. All authors contributed to the article and approved the submitted version.
